# Pharmacogenetic and pharmacogenomic discovery strategies

**DOI:** 10.20517/cdr.2018.008

**Published:** 2019-06-19

**Authors:** Concetta Crisafulli, Petronilla Daniela Romeo, Marco Calabrò, Ludovica Martina Epasto, Saverio Alberti

**Affiliations:** ^1^Department of Biomedical Sciences - BIOMORF, University of Messina, via Consolare Valeria, 98125 Messina, Italy.; ^2^Unit of Medical Genetics, University of Messina, via Consolare Valeria, 98125 Messina, Italy.

**Keywords:** Pharmacogenetics, pharmacogenomics, cancer, next-generation sequencing, genomic variants

## Abstract

Genetic/genomic profiling at a single-patient level is expected to provide critical information for determining inter-individual drug toxicity and potential efficacy in cancer therapy. A better definition of cancer subtypes at a molecular level, may correspondingly complement such pharmacogenetic and pharmacogenomic approaches, for more effective personalized treatments. Current pharmacogenetic/pharmacogenomic strategies are largely based on the identification of known polymorphisms, thus limiting the discovery of novel or rarer genetic variants. Recent improvements in cost and throughput of next generation sequencing (NGS) are now making whole-genome profiling a plausible alternative for clinical procedures. Beyond classical pharmacogenetic/pharmacogenomic traits for drug metabolism, NGS screening programs of cancer genomes may lead to the identification of novel cancer-driving mutations. These may not only constitute novel therapeutic targets, but also effector determinants for metabolic pathways linked to drug metabolism. An additional advantage is that cancer NGS profiling is now leading to discovering targetable mutations, e.g., in glioblastomas and pancreatic cancers, which were originally discovered in other tumor types, thus allowing for effective repurposing of active drugs already on the market.

## Introduction

Advances in cancer treatment over the last decades have more and more relied on identifying therapy-responsive subgroups on the basis of evolving tumor subgroup classifications^[1]^, as based on pathological and clinical parameters^[2]^, tumor stage^[3]^, cancer biomarkers^[4,5]^ and cancer drivers^[6-14]^, including both somatic and hereditary epigenetic changes^[15,16]^. However, critical limitations remain in our ability to predict therapeutic efficacy and toxicity for tumor subgroups which appear homogeneous by all the above criteria, such target remaining an unmet medical need^[17]^.

**Figure 1:**
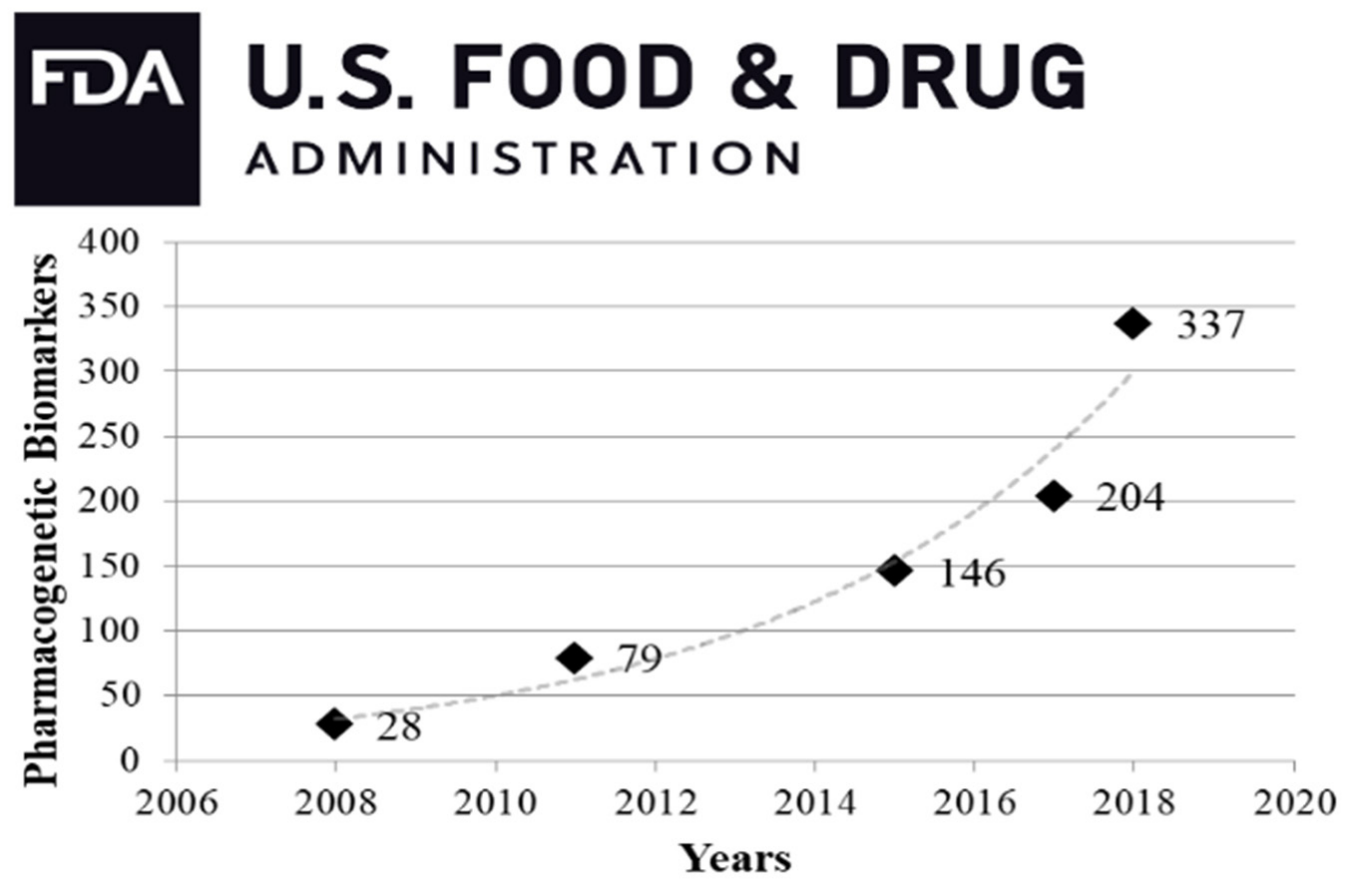
Pharmacogenetic biomarkers in FDA drug labels over the last decade (www.fda.gov/BiologicsBloodVaccines/DevelopmentApprovalProcess/BiologicalApprovalsbyYear/ucm596371.htm) has undergone an exponential increase. It should be noted that this data-set covers both somatic mutations and hereditary variants, together with genes that indirectly affect pharmacokinetics through drug-drug interactions

Over the past several years, there has been a major shift in cancer diagnostics from physical examination/*in vivo* imaging/histopathological analysis to assessment of tumor biomarkers, cancer drivers and targetable genomic mutations. Genetic variation in drug response is well documented and influences treatment efficacy and/or toxicity^[17]^. Numerous genomic variants are increasingly recognized as both potential therapeutic targets and drug metabolism modifiers^[18,19]^, thus gaining recognition by regulatory agencies [Figure 1, Supplementary Table 1]. The concept of precision/personalized treatment is more and more supported by these complementary strategies, so that clinical decisions are adjusted on the basis of each patient’s genetic background, as complemented by detailed information on the patient’s tumor^[20,21]^.

## Pharmacogenetic and pharmacogenomic discovery strategies

Pharmacogenetics and pharmacogenomics constitute a branch of molecular medicine focused on the study of human genome in the context of therapeutic decision-making^[22]^. Pharmacogenetics is the study of inherited variations in the DNA sequence of known genes, e.g., involved in drug metabolic pathways, which can affect individual responses to drugs, both in terms of therapeutic responses and adverse effects^[23]^. Pharmacogenomics is the study of acquired and inherited genetic differences in relation to drug response and drug behavior, through a systematic examination of DNA sequence at the level of the entire genome^[17]^.

Germline mutations may help to predict alterations in drug-metabolism-related genes (e.g., CYP450) and pathways^[24]^. This can aid in correspondingly adjusting for systemic drug exposure, with the aim of minimizing drug toxicity and improving disease outcome^[17,25,26]^. Drug’ efficacy and toxicity rely on complex interactions between different gene-encoded metabolic pathways and environmental factors. Highly, complementary approaches are required to dissect such complexity [Table 1].

**Table 1:** Discovery strategies for novel pharmacogenetic and pharmacogenomic traits

Advantages	Disadvantages
Candidate Polymorphism Analysis	
1. Rapid execution of the assay	1. Polymorphisms need to have strong effects toward the phenotype
2. Focus on genes likely involved in treatment response and toxicity	2. It is based on validated knowledge, ad may miss potentially-involved unknown genes
	3. It ignores *de-novo* mutations in target genes
Pathway analysis	
1. Focus on pathways, downstream the gene(s) of interest, that are highly likely to be involved in the drug action	1. It may miss potentially involved, but still unidentified, signaling cascades
2. It highlights whole signaling cascades, for higher sensitivity for genes with smaller phenotypic effects	2. Data analysis is complex given the interplay of multiple interacting genes
3. It can identify new polymorphisms or new genes within a given pathway	3. It requires investigating large sample case-series
4. More likely to explain inter-individual variation in drug response	
“Whole genome strategies”	
1. They provide a complete gene- or protein- expression profile (tumor or individual)	1. The lack of hypothesis-driven analyses may increase the risk of false positives
2. They provide information on novel associations	2. Complex data management and analysis procedures are required
3. They generate large amounts of data	3. Costs and complexity still high for the clinics
4. Useful in predicting tumor response	

### Cancer-driving somatic DNA mutations and inherited DNA variants that may impact on pharmacogenetic and pharmacogenomic strategies

Cancer-causing DNA alterations, such as somatic DNA mutations and inherited DNA variants, are not a direct focus of pharmacogenetic and pharmacogenomic studies. However, mutated cancer drivers are becoming more and more actionable targets for therapy. They can also affect key metabolic pathways that may modify drug pharmacokinetics and pharmacodynamics. As such, they can play a key role in pharmacogenetic and pharmacogenomic discovery.

Key examples are DNA-damage response pathways (ATM, CHEK2, BRIP1, BRCA1, BRCA2, PALB2), which are associated with an increase in breast cancer risk^[27]^. In particular, germline pathogenic variants in BARD1, BRCA1, BRCA2, PALB2 and RAD51D are associated with high risk (odds ratio > 5.0) for triple-negative breast cancer (TNBC)^[28,29]^. BRCA1, BRCA2, PALB2 variants are also associated with increased risk of ovarian^[30]^ and other cancers^[31,32]^. Pathogenic variants in BRIP1, RAD51C and TP53^[33]^ are associated with moderate risk (odds ratio > 2) for TNBC, whereby hereditary pathogenic variants are detected in 12.0% of TNBC^[28]^.

Corresponding mutations in the *BRCA1*, *BRCA2*, *PALB2* genes are detected in a fraction of sporadic breast and ovarian cancer^[34]^. Notably, mutations in BRCA1, BRCA2 are associated to better therapeutic response to PARP inhibitors^[35]^, making such mutations effective tools for therapy choice. Other, cancer-associated somatic mutations, e.g., the mutations at codons 12 or 13 in the *Ki-RAS* gene, predict lack of response to EGFR-targeted therapy in colon cancer^[36]^. Mutations in the EGFR kinase ATP-binding pocket mandate choosing specific tyrosine kinase inhibitors^[37]^. Of interest, such EGFR mutants also are predictors of response to unrelated therapy^[38]^.

It should be noted that mutated cancer-driving genes may play a key role as metabolic-pathway modifiers. Examples are mutations in *TP53*, which differentially impact on metabolic pathways and apoptotic responses, thus modifying the impact of anticancer chemotherapy. Not surprisingly, *TP53* mutations are predictive of poor response to therapy^[39]^. Activation of transcription of c-Myc impacts on main metabolic pathways, on ribosomal biogenesis, and on lipid metabolism^[40]^. *In vivo* efficacy of the Bcl-2 antagonist ABT-737 depends on c-Myc activation^[41]^. Furthermore, c-myc/p53 interactions determine sensitivity of colon cancer to 5-Fluorouracil^[42]^. Mutations of the *PIK3CA* gene are predictors of response to AKT and mTOR inhibitors^[43]^. Hence, taking into account the above caveats, cancer drivers can constitute rather attractive targets for pharmacogenetic investigation.

As whole-genome information is rapidly accumulating [Figure 1], it is likely that more and more genetic factors, or clusters of them, will be discovered that may affect both tumor progression, drug pharmacodynamics and overall response to therapy. Such cross-feeding amid convergent research fields is expected to foster better knowledge about gene-gene interactions *vs.* therapeutic drug metabolism.

### Candidate polymorphism search

This type of analysis seeks polymorphic DNA sequences within specific genes, known to impact on the pharmacokinetics or pharmacodynamics of a compound. Such information may aid in the selection among different therapeutic strategies. When sufficiently large clinical data are available, they may also aid in drug dosage selection.

A potential functional impact of genes mapping near polymorphic sites can be explored through gene silencing. Gene silencing can be obtained through RNA interference via short hairpin RNA (shRNA; preceding methods relied on siRNA)^[44-46]^. shRNA have been proposed for use as pharmacological compounds^[47]^. More recent applications include Clustered Regularly Interspaced Short Palindromic Repeats (CRISPR) technology, which can be utilized to ablate target transcription factors, chromatin-modifying factors, and noncoding RNA, thus providing powerful instruments for gene silencing purposes^[48-51]^.

### Candidate pathway strategy

This strategy covers a wider range than the previous one. It still retains some of the limitation of the candidate-gene approach, namely the need of previous knowledge regarding the biological cascades correlated to a compound action. However, the pathway approach extends such an analysis to larger numbers of related genes, whose altered function may impact on treatment efficacy. This approach may add significant chances to explain inter-individual variation in drug efficacy/toxicity, as it includes evaluation of potential epistatic effects and of the influence of other cis-regulatory elements^[51]^. These studies may also discover gene-gene interactions as pharmacological targets.

Newly added research strategies in this field are based on the concept of synthetic lethality. A synthetic lethality event occurs when two or more genes are simultaneously perturbed and exposure to a drug results in cellular or organism death/impairment^[52]^. Large-scale analysis in yeast for interactions among orthologs of human tumor suppressor genes allowed to evaluate thousands of genotype-drug combinations, that were then transferred to cancer cells *in vitro*. This resulted in the identification of networks of conserved, synthetic lethal interactions. Among them, the interaction of topoisomerases with RAD17 and of checkpoint kinases with BLM were validated by patient survival data^[53]^. These techniques inform on gene-gene and gene-drug dependencies, taking advantage of better knowledge on control gene pathways^[54]^, candidate for targeted drug design^[51]^. PARP inhibitor efficacy in *BRCA1* and *BRCA2* mutation carriers is limited by inherent and acquired resistance. By using synthetic lethality in combination with PARP inhibitors, BRCA2-mutant cells were found to be dependent on base excision repair, Atr activation and mRNA splicing. Subsequently, FEN1 and APEX2 were identified as BRCA2 synthetic lethal targets^[55]^.

### Whole-genome strategies

The previously described approaches suffer from a critical limitation, i.e., they only focus on pre-specified sets of genes (knowledge-based), thereby ignoring potential candidates whose effect has not been linked, yet, to treatment efficacy/toxicity. This limitation can be overcome by global strategies, whereby the focus of the search is extended to the entire genome, transcriptome or proteome of an individual or group of individuals. Since the completion of the human genome project and the early efforts to map human genetic variations, tools were developed to evaluate the relationship between these alterations and drug efficacy in an essentially unbiased fashion. Using high-resolution genotyping platforms, genome-wide association studies (GWAS) were rapidly conducted on nearly all common cancers. Although hundreds of statistically robust risk variants, largely in the form of single-nucleotide polymorphisms (SNP), were identified, each genetic variant was mostly associated with a modest increase in disease risk (relative risk ≤ 1.5). Moreover, as ≈90% of risk variants reside in noncoding introns, causal factors associated to most risk loci have remained elusive^[56]^. Albeit a useful tool for investigation and discovery of new genes, the use of whole-genome strategies in clinical practice is restricted by high costs, labor intensity and complex interpretation of the data. Thermodynamic limitations^[57]^ add to the high amount of noise associated with this system.

## The next-generation sequencing era

**Figure 2:**
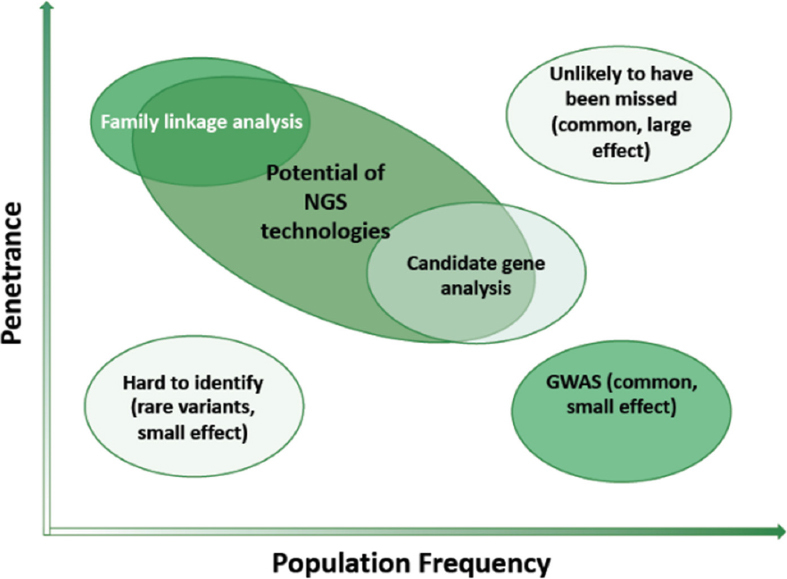
The efficiency of discovery of novel pharmacogenetic/pharmacogenomic biomarkers, modified from^[27]^. The combined expected impact of genomic variants on disease occurrence and genomic diagnostics is indicated (light green: low; deep green: high)

Better knowledge on cancer drivers^[7-14]^, on their use as therapy-target molecules^[13,58]^ and on their interaction with drug efficacy/ toxicity determinants^[9,13,14,39,41,59]^ has reached milestone successes^[39,59]^. However, current strategies remain largely based on known polymorphisms (from the human genome project), thus limiting the discovery of novel/rarer mutations [Figure 2]^[60]^.

### Pharmacogenetic and pharmacogenomic biomarkers

FDA defines genomic biomarkers as a measurable DNA and/or RNA characteristic that can be used as an indicator of either normal biologic processes, pathogenic phenomena, and/or response to therapeutic or other interventions. Frequently used genomic biomarkers include SNP and DNA segments copy number variations (CNV) (www.fda.gov/ucm/groups/fdagov-public/@fdagov-drugs-gen/documents/document/ucm073162.pdf). Applications of genomic biomarkers in pharmacogenomics and pharmacogenetics are more and more frequent (www.fda.gov/ucm/groups/fdagov-public/@fdagov-drugs-gen/documents/document/ucm073162.pdf). Currently, though, no specific recommendation for the use of genomic biomarkers’ have been established. Somewhat general guidelines for the use of biomarkers can be found at (www.fda.gov/ucm/groups/fdagov-public/@fdagov-drugs-gen/documents/document/ucm628118.pdf).

### Whole-genome profiling

**Figure 3:**
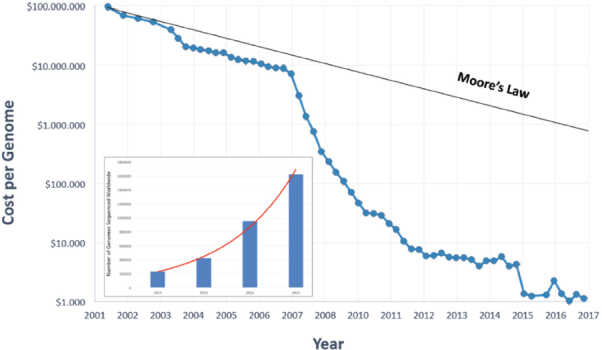
NGS costs per whole genome sequencing. Inset: number of whole genomes sequenced worldwide, as a compilation from publicly available genome-sequencing projects (as modified from www.genome.gov/images/content/costpergenome_2017.jpg)

A global profiling of each individual may critically help identify determinants of predisposition to drug toxicity, as well as to drug efficacy^[61]^. Improvements in cost and throughput [Figure 3] have recently surpassed competing development of leading microelectronic technologies (Moore’s law), and have made next-generation sequencing (NGS) approaches realistic and feasible [Supplementary Table 2 and 3].

Data from publicly available genome-sequencing project data indicate fitting with an exponential growth curve over time (Figure 3, inset; as modified from cdn.static economist.com/sites/default/files/images/2014/11/articles/main/20151101_stc002_595.png). Correspondingly, information on the complexity of cancer states and on tumor progression trajectories are being rapidly obtained^[21,61]^.

Such efforts have already led to a marked increase in the number of targeted therapies approved for the treatment of patients with specific types of malignancies harboring specific types of sequence alterations^[62]^ [Supplementary Table 1].

### NGS applications in discovery strategies

The field of pharmacogenetics/pharmacogenomics has uncovered an abundance of actionable, clinically relevant markers, through distinct, complementary approaches.

#### Hotspot panels

Hotspot panels, examples of which are listed in Supplementary Table 4, target frequently mutated gene stretches (hotspots), which are expected to bear diagnostic/prognostic significance. Such panels have been successfully used to guide cancer treatment, e.g., for lung cancer^[63]^. Most hotspot panels detect somatic mutations in tumor DNA. Previous comments on the distiction between cancer drivers/therapy targets *vs.* pharmacogenetic/pharmacogenomic determinants also apply here. As these panels only analyze known, pre-determined areas within specific genes, they are not amenable to the discovery of novel mutations. Even if agreement on guidelines that should be followed in clinical practice while using testing panels has yet to be reached^[64]^, several consortia have been created to aid in the use of pharmacogenetic data (e.g., Clinical Pharmacogenetics Implementation Consortium)^[65-67]^.

#### Actionable gene panels

Similar to the hotspot ones, these panels focus on specific genes. However, they include all exons of the targeted genes, so that alterations not explicitly included in hotspot panels can also be investigated. Panels of this type have been developed for melanoma, colon and gastric cancer. While these can be considered an evolution from hotspot panels, they still do not guarantee complete coverage of the target genes (no intronic or regulatory sequences are included) and remain anchored to preexisting knowledge (only genes already known to be involved in drugs’ action are included).

#### Disease-focused panels

Similarly to the actionable panels, disease-focused panels include sets of genes (exomes) known to be involved in a specific disease. These are mainly used as a screening panel to evaluate the hereditary risk of developing specific tumors, e.g., BRCA-1, -2-driven breast and ovarian cancers. This type of panel is more useful for screening programs and in early/preventive management of cancer.

#### Comprehensive panels

The difficulty in recruiting adequately large sample groups of homogeneous tumors within realistic time-frames rarely justifies the costs of production of different disease focused panels. This has led life science companies to create larger, more comprehensive panels in which multiple genes that correlate with multiple diseases are included. These panels can include thousands of genes (exons only) which are selected among the ones that are most commonly tested. Like other targeted panels, comprehensive panels depend on pre-existing knowledge on the correlation between genes and the investigated phenotype.

#### Validated panels

As of November 27th, 2018, ~1677 NGS-based clinical tests for cancer were available across the world (search terms “NGS” AND “Cancer” in the NIH Genetic Testing Registry, website www.ncbi.nlm.nih.gov).

#### Whole exome sequencing

Despite the ever-increasing number of genes that are included in targeted panels, the risk of not including elements that are possibly correlated with the investigated phenotype remains high. To overcome this limitation increasing numbers of researchers resort to whole exome sequencing (WES). This approach allows to identify both known and unknown alterations that occur within coding regions. Some groups estimate that WES may cover ≈85% of disease-related variants^[63]^. This estimate should be taken with great caution, as approximately 98% of the genome remains uncovered. Such regions include regulatory elements, non-coding mRNA, splice sites, anchor sites to the nuclear membrane, chromatin modifiers, and other regions we simply don’t know enough about.

#### Whole genome sequencing

Whole genome sequencing (WGS) provides a global coverage of the human genome. This approach represents the last and most comprehensive step in genomic analysis. The feasibility of this approach in recent years was aided by the introduction of higher-processivity NGS technologies (higher speed, lower costs). The main limitation of this approach is essentially related to the complexity of data post-analysis, and related statistics, with considerable risk of both false positives and false negatives.

#### RNA sequencing

With a coverage comparable to that of WES, RNA sequencing profiles the transcriptome of a tumor. Differently from WES, whose target is the subject’s DNA, RNA provides a dynamic/functional image of a cancer. It has the advantage of detecting the presence of fusion (onco)genes, which are often found in cancer cases^[8,68,69]^. It may aid clinicians in the choice of drug therapy and basic science for the identification of novel potential drug targets.

#### Chromatin immunoprecipitation sequencing

This approach is normally used to understand DNA-protein binding interactions, e.g., regulatory protein-binding sites within a DNA region. This also has the capability to detect epigenetic alterations^[70,71]^. The use of chromatin immunoprecipitation sequencing in cancer therapy is limited, but it has proven useful in stratifying breast cancer subtypes for treatment^[72]^.

## Top-ranking NGS technologies

The birth of NGS sequencing can be placed in 2005, when 454 Life Sciences launched their sequencing-by-synthesis tool^[73]^, as the first instrument of a 2nd generation sequencing technology. Since then, many companies have developed different NGS products, which are usually based on different, proprietary procedures and base-detection chemistry. Current best-ranking technologies, comparative performance and preferential platform applications are presented in Supplementary Table 2 and Supplementary Table 3.

Albeit reference technologies vary widely across NGS platforms, workflows share several steps: (1) DNA extraction; (2) library preparation, with addition of adaptors and barcodes/indexes; (3) template preparation, either by bridge amplification or emulsion PCR; (4) automated sequencing [Supplementary Table 3].

The pioneer Roche/454 (www.sequencing.roche.com) sequencing used pyrosequencing, as based on the detection of pyrophosphate released after nucleotide incorporation in newly synthesized DNA. Currently, this platform is rarely utilized, because of long run times and high costs.

The Supported Oligonucleotide Ligation and Detection (SOLiD) platform (www.thermofisher.com), is a short-read sequencing technology based on ligation. DNA fragments are ligated to oligonucleotide adapters, attached to beads, and clonally amplified by emulsion PCR. Beads with clonally amplified template are immobilized onto a derivatized-glass flow-cell surface, and sequencing is started by annealing a primer oligonucleotide complementary to the adapter^[74]^. At each cycle the complementary strand is removed and a new sequencing cycle starts at the position n-1 of the template. Sequencing cycles are repeated until each base is sequenced twice. The recovered data from the color space can be translated to letters of DNA bases and the sequence of the DNA fragment is assembled^[75]^.

The IonTorrent platform (www.thermofisher.com) is based on sequencing by synthesis and the detection is based on solid state pH meters, which measure the hydrogen ions that are released during DNA polymerization.

Illumina (www.emea.illumina.com) currently occupies the most prominent segment of the NGS market. This technology is based on sequencing by synthesis of template-complementary strands and on fluorescence-based detection of reversibly blocked terminator nucleotides^[76]^.

All technologies above (2nd generation sequencing) require demanding protocols with serial PCR steps that results in an increased time of processing and cost. Moreover, genome regions with high density of repeats are difficult to decipher when using short reads^[75]^. With the entry in the market of 3rd generation sequencers, several such hurdles have been overcome. To date, popular platforms are the Pacific Biosciences and the Oxford Nanopore technologies.

Pacific Biosciences use single molecule real-time (SMRT) technology. Library preparation leads to a closed circular DNA molecule by ligating an adaptor molecule to both ends of the target DNA molecule to be sequenced. The circular DNA molecule is then loaded into a cell containing 150,000 zeptolitre wells (ZMW)^[77,78]^. Each ZMW contains a DNA polymerase attached to the bottom and the target DNA fragment for sequencing. During the sequencing reaction, the DNA fragment is labeled by the DNA polymerase with fluorescent nucleotides and the corresponding emitted signal is recorded (www.pacb.com)^[75]^.

The Nanopore technology identifies DNA bases by measuring changes in electric conductivity generated as DNA strands pass through a biological pore. The chemical differences of each base would result, in theory, in detectably altered current flow through the pore. During the library preparation step, fragmented DNA is repaired using a PreCR step. Two adaptors are then added to the DNA, a Y adapter and a hairpin adaptor. A motor protein unzips the double stranded DNA at the Y adapter and feeds the DNA as a single strand through the nanopore (www.nanoporetech.com)^[77,78]^.

The BGI BGISEQ-500 sequencer uses a Probe-Anchor Synthesis (cPAS) and a DNA Nanoballs (DNB) technology. The cPAS chemistry works by incorporating a fluorescent probe into a DNA anchor on the DNB, followed by high-resolution digital imaging. This combination of linear amplification and DNB technology reduces the error rate while enhancing the signal. In addition, the size of the DNB is controlled in such a way that only one DNB is bound per active site (www.bgi.com).

## Validation of NGS findings

The rapid evolution of NGS technology keeps enhancing throughput and reducing run time and costs. Thus, NGS appears ready to offer opportunities for implementation in clinical procedures. On the other hand, though, the inevitably labor-intensive and time-demanding clinical experimentation is vastly lagging behind, thus fostering ‘home-made’ adoption of genomic tests (www.23andme.com/en-int/), in the virtual absence of experimentally-validated guidelines. Genetic information demands may create further health care disparities because of the high cost of these technologies^[27]^ and even pose health care risks if results are prematurely translated to the consumer market outside of regulatory protection^[27]^ (www.genomeweb.com/dxpgx/fda-warns-consumer-genomics-firms-illumina-selling-unapproved-dx-products#.XBLPfBNKi-U). As such, these practices are devoid of validated medical status (www.nytimes.com/2013/11/26/business/fda-demands-a-halt-to-a-dna-test-kits-marketing.html).

## Guidance from regulatory agencies

FDA and EMA have established recommendations to adopt pharmacogenetic and pharmacogenomic methods in research and diagnostics. Adequately numbered studies are highly recommended for achieving meaningful screening power, particularly when target mutations are rare (www.fda.gov/ucm/groups/fdagov-public/@fdagov-meddev-gen/documents/document/ucm071075.pdf). Regulatory agencies correspondingly urge to create public databases, that would include global pharmacogenetic data to provide key scientific input to both basic and clinical research (www.fda.gov/ucm/groups/fdagov-public/@fdagov-meddev-gen/documents/document/ucm509837.pdf).

Pharmacogenetic and pharmacogenomic tests will play ever more important roles in efforts to prevent adverse drug reactions. In this case, drug-metabolizing enzymes, drug transporters and drug targets are potential target genes, as they may alter the drug action or metabolism. HLA typing itself may be considered a biomarker of drug response, as it is associated to distinct haplotypes/populations with different prevalence of specific mutations in actionable genes. According to EMA, the evaluation of HLA can be carried out in drug developmental programs, in order to discover new predictive HLA biomarkers. The use of whole exome sequencing for the HLA region is strongly recommended by EMA, and is proposed to become the gold standard for HLA typing (www.ema.europa.eu/documents/scientific-guideline/guideline-good-pharmacogenomic-practice-first-version_en.pdf). Technical recommendations have also been issued, as EMA recommends that the technical predictive value of NGS should be at least 99.9%. In germline genetics, a minimum coverage of > 30× is desirable. A higher one should be pursued if a genetic variant is uncommon (www.ema.europa.eu/documents/scientific-guideline/guideline-good-pharmacogenomic-practice-first-version_en.pdf). It is expected, though, that improvements in current technologies will rapidly superseed these thresholds.

## Impact of NGS - successful strategies and proof-of-concept achievements

Rapid progress in deciphering cancer genomes is being achieved through ongoing international efforts, including The Cancer Genome Atlas and the International Cancer Genome Consortium. These collaborative efforts aim at sequencing up to 500 clinically well-annotated tumor groups, with the projected generation of an enormous amount of genomic data. To date, the Cancer Genome Atlas has already published initial analyses of glioblastomas and ovarian, colorectal, and breast cancers, identifying a number of deregulated genes that may set the stage for the development of targeted therapies and for associated pharmacogenetic/ pharmacogenomic biomarkers.

The shift to knowledge-driven cancer treatment requires novel classification strategies of cancer endotypes^[1]^. Improvements in 2nd and 3rd generation NGS [Supplementary Table 2 and 3] have paved the way for the use of pharmacogenomics testing at a whole-genome level^[61]^.

Clinical oncology utilization of current knowledge on cancer hereditary genetics (ascopubs.org/toc/edbk/current) largely focuses on highly penetrant genes, which can account for a considerable fraction of a given cancer risk. Main examples comprise mismatch DNA repair genes, for Lynch syndrome, BRCA-1, -2, PALB2 for ovarian, breast and prostate cancer, *TP53* for Li-Fraumeni syndrome (www.cancer.gov/about-cancer/causes-prevention/genetics/genetic-testing-fact-sheet#q4) and are extensively treated in other chapters of this journal issue.

Cancer genomics can extensively identify and quantify cancer inherited risk^[27]^. Tumor DNA sequencing and comparison to the germline genome may identify variants associated with hereditary predisposition to cancer. Such an ability to achieve high-throughput genotyping often surpasses, though, our current ability to interpret and appropriately apply the vast amounts of data that are generated. Although known hereditary cancer susceptibility syndromes are more than one hundred, mutations in high-penetrance genes explain only a fraction of the heritability of human cancers [Figure 2]^[79]^.

GWAS have been conducted on nearly all common cancers. However, given the modest effect size for most risk variants, the clinical utility of genomic profiling for risk stratification based on GWAS data has been limited. A large international consortium study led to the identification of 49 new loci for breast cancer, 26 for prostate cancer, and 8 for ovarian cancer. Extension of such approaches may allow better genetic susceptibility models of cancer risk.

Despite such extensive research, only approximately 30% of familial breast cancer risk is explained by known genetic factors. WES in affected family members from 13 breast cancer families identified two families with mutations in XRCC2, including a protein-truncating change and a probable deleterious missense mutation. Another Fanconi pathway gene, SLX4 was found in only one of ≈700 BRCA-negative breast cancer kindreds. Most recently, a large pooled NGS study focusing on DNA repair pathways identified mutations in the p53-inducible protein phosphatase PPM1D as occurring mosaically in individuals with predisposition to breast and ovarian cancers. SNP-associated risk analysis identified a frameshift mutation in the BRIP1 Fanconi pathway gene, with an odds ratio of 8.1 for ovarian cancer. As indicated above, the identification of genes that are associated with homologous recombination DNA-repair has a role for targeted therapies, such as poly (ADP-ribose) polymerase inhibitors. WGS in families with multiple adenomas and/or colorectal cancer recently identified heterozygous POLE and POLD1 germline variations. GREM1 is the first gene to have been implicated in the genetic etiology of hereditary mixed polyposis syndrome. In prostate cancer, NGS of linkage regions on chromosome 17 helped to identify a mutation in the homeobox gene HOXB13 as a prostate cancer driver and potential therapy target.

## Repositioning of cancer-driving mutations and of corresponding therapy targets

NGS screening programs of cancer genomes have surprisingly led to the identification of cancer-driving mutations in tumor types that were essentially unrelated to the ones under investigations. As several driver mutations are therapy targets of drugs already in the clinics, this is rapidly leading to the repurposing of drugs that are already known to be effective against other tumor types. These serendipitous findings are now leading to a research effort that promises to be effective in adding new molecular-target therapies to tumor types often in large need of impactful treatments, such as glioblastomas and pancreatic cancer^[21,27,80]^.

One of the first successes of NGS in this respect was the use of WES to identify risk factors that are linked to pancreatic cancer, such as PALB2 and ATM^[27]^. Other examples are the E318K MITF variant, which as indicated above associates to melanoma, but was subsequently found to associate to renal cancer; the POLE and POLD1 germline variations, which are associated to colon cancer, however mutations in POLD1 also increase endometrial cancer risk; somatic mutations in BAP1, which were first identified in mesothelioma and uveal and cutaneous melanomas, were subsequently associated to renal cell cancer.

## Quality checks

NGS protocols require highly standardized procedures for pre-analytic, analytic, and post-analytic processes. Such requirements follow the guidelines for clinical laboratory tests according to the Clinical Laboratory Improvement Amendments (CLIA) (www.cdc.gov/CLIA).

Although efficient and cost-effective, NGS has the disadvantages of high error rates and short read lengths, enrichment of rare variants, and a large proportion of missing values. A comparison of the accuracy and completeness of variant calling for two commonly used sequencing platforms found that although both technologies achieved a relatively high concordance (88%) for unique single-nucleotide variants, concordance for indel detection was only 27%.

The American College of Medical Genetics and Genomics (ACMG) has correspondingly developed a position statement for the detection of germline mutations by whole exome and genome sequencing and for the validation of NGS methods and platforms, through monitoring NGS testing, data interpretation and reporting^[81]^. Similar quality assurance guidelines for NGS in diagnostic pathology are being established in Europe^[82]^. The ACMG and the Association for Molecular Pathology (AMP) have jointly published recent laboratory standards and guidelines^[83]^.

Accordingly, any new alteration found through NGS (WES, WGS, RNA-Seq) needs to be confirmed through another method. The main method is Sanger sequencing^[84]^. Other validation methods have been proposed, such as orthogonal NGS, i.e., the parallel sequencing of target genomes using two different NGS platforms^[84,85]^. As for clinical applications, pre-analytic sample quality, such as tumor content of specimens, DNA integrity and yield, still are key to robust findings and must be emphasized to avoid pre-laboratory errors. Uncertainty remains on best practices regarding identification of indels and of epigenetic changes^[86,87]^, which remain technically demanding for current NGS technologies.

## NGS bottlenecks

As previously mentioned, storing, managing, analyzing, and interpreting genome-wide data are now rapidly becoming the bottleneck of NGS analysis procedures. Caution is also required in data interpretation, as each distinct NGS procedure has sequencing error biases [Supplementary Table 3]^[88]^. The occurrence of CNVs, as that of large insertions, causes inherent difficulties (suboptimal sequence coverage and mapping quality) in NGS readings, which relate to the short-read-based nature of most NGS platforms^[88]^. Recent technological advances aim at tackling this issue. High-homology regions also lead to inappropriate alignment of the reads^[84,89]^, and this is particularly important in pharmacogenetic tests for pharmacokinetics genes, as CYP isoforms share a large sequence similarity. Also, the presence of a too high or too low GC content may affect the accessibility of gene regions^[84]^. It has been estimated that more than 12% of the human exome contains “difficult or unable to be analyzed” regions for NGS because of the presence of multiple, highly homologous genes^[89]^.

Another important issue is related to the creation of reference databases. Several efforts have been initiated to reach this aim, such as the National Center for Biotechnology Information-sponsored data repository ClinVar. Other large pharmacogenomic data repositories such as The Pharmacogenomics Knowledge Base (PharmGKB) developed by Stanford University **(**www.pharmgkb.org**)** and other data sets, including those available from the National Institutes of Health (NIH) GWAS collection (www.ncbi.nlm.nih.gov/snp), through the use of electronic medical records^[90]^ have made it possible to computationally analyze personal genomes for potential translation of pharmacogenomics into clinical practice.

## Single-cell omics developments

Neoplasias are heterogeneous diseases that interact with complex microenvironments and batch analyses inevitably “average” such target heterogeneity. Genomic sequencing at the single-cell level bears the potential of identifying both distinct genetic cancer drivers and control networks/ druggable targets in heterogeneous neoplastic populations^[91]^. Single-cell genomic assays faithfully detect somatic mitochondrial DNA mutations, track cellular relationships and hierarchies, and enable definition of clonal architecture in human cancer.

Combined single-cell RNA sequencing and genotyping can profile distinct subclones of the same tumor. This allows to identify dysregulated transcriptional programs driven by potential drug-targetable genes, with implications for targeted medicine. Single-cell DNA and RNA sequencing during neoadjuvant chemotherapy can identify patients in which treatment leads to clonal extinction *vs.* those in which clones persist after treatment. Notably, this analysis can reveal pre-existing resistant genotypes that could become pharmacogenomic discovery targets^[92]^. Improvements of single-cell RNA-sequencing procedures are ongoing to correspondingly enhance the efficiency of such screening procedures.

Corresponding strategies can be applied to single-cell profiling and functional screening of long non-coding RNA^[93]^. These approaches will critically allow extending the identification of candidate diagnostic control networks and therapeutic targets beyond the protein-coding regions of the genome.

Single cell proteomics by flow cytometry allow to track and analyse signalling events in individual cancer cells and to create signalling network maps in each cell, to identify both common fundamental regulatory themes and population heterogeneity. This can identify pathways that are activated in therapy-resistant cells and can provide predictive, actionable cancer targets^[94]^. Additional data can be provided by mass cytometry, for high-dimensional, quantitative analysis of the effects of bioactive molecules on cell populations at single-cell resolution. Correlation of proteomic, transcriptomic and mutagenomic profiles with intracellular signaling molecules allows to correlate biological functions, such as metabolism, survival, DNA damage, cell cycle and apoptosis, to provide determination of network states of heterogeneous populations of individual cancer cells. Improvements in bioinformatic single-cell data comparison will be instrumental in taking the greatest advantage of single-cell measurements^[95]^.

## Conclusions and future directions

Our current knowledge of rare and common somatic/genetic variants associated with cancer risk, pharmacological treatment and disease outcome has led to significant progress, as well as to a number of challenges associated with the clinical translation of these discoveries. Improved pharmacogenetic and pharmacogenomic knowledge will extend known associations of genetic variabilily with drug responses. Key limitations of traditional approaches, i.e., exclusive focus on already known sets of genes, will most likely be overcome by global strategies, whereby the focus of the search will be extended to the entire genome, transcriptome or proteome of an individual or groups of individuals.

Better characterization of a cancer entity will doubtless contribute to improved therapy. Additional knowledge will be gathered by novel research strategies, such as those based on the concept of synthetic lethality. A synthetic lethality event occurs when two or more genes are simultaneously perturbed and exposure to a drug results in cellular or organism death/impairment. Large-scale analysis in yeast and human cells have already resulted in the identification of actionable networks of conserved, synthetic lethal interactions.

An additional level of cancer pharmacogenomics will be reached by single cell omics analyses. These will obtain information from individual cancer cells, to identify both common regulatory themes as well as population heterogeneity. This is expected to lead to the identification of subpopulations of therapy-resistant cells and of the corresponding control pathways^[94]^.

A key role is all the analyses above will be provided by next-generation bioinformatic approaches, which will be instrumental in taking the greatest advantage of whole-genome sequencing at population levels and of single-cell measurements^[95]^.

According to National Comprehensive Cancer Network, NGS-based pharmacogenetic/pharmacogenomic assays still are qualified as second-tier tests. The growing body of pharmacogenomic data may soon allow to go well beyond such a stage and to provide strong means to guide clinicians in the selection of safer and more effective therapies^[17]^.

## Declarations

### Copyright

© The Author(s) 2022.
