# Supplementary material for: Pharmacogenetic and pharmacogenomic discovery strategies
Source: Cancer Drug Resist. 2019 Jun 19;2(2):225–41. doi: 10.20517/cdr.2018.008 (PMC8992635; doi:10.20517/cdr.2018.008)
Supplement: Supplementary file 1 [file cdr-2-225-SupplementaryMaterials.pdf]

Supplementary Table 1: Pharmacogenetic Biomarkers in Cancer.

Drug	Therapeutic Area	Biomarker
**Abemaciclib** (http://www.accessdata.fda.gov/scripts/cder/daf/index.cfm?event=overview.process&varApplNo=208716)	Oncology	ER [a,b]<br>ERBB2 (HER2)
**Ado-Trastuzumab Emtansine** (http://www.accessdata.fda.gov/scripts/cder/daf/index.cfm?event=overview.process&varApplNo=125427)	Oncology	ERBB2 (HER2)
**Afatinib** (http://www.accessdata.fda.gov/scripts/cder/daf/index.cfm?event=overview.process&varApplNo=201292)	Oncology	EGFR
**Alectinib** (http://www.accessdata.fda.gov/scripts/cder/daf/index.cfm?event=overview.process&varApplNo=208434)	Oncology	ALK
**Anastrozole** (http://www.accessdata.fda.gov/scripts/cder/daf/index.cfm?event=overview.process&varApplNo=020541)	Oncology	ER, PgR [c]
**Arsenic Trioxide** (http://www.accessdata.fda.gov/scripts/cder/daf/index.cfm?event=overview.process&varApplNo=021248)	Oncology	PML-RARα
**Atezolizumab** (http://www.accessdata.fda.gov/scripts/cder/daf/index.cfm?event=overview.process&varApplNo=761034)	Oncology	CD274 (PD-L1)
**Avelumab** (http://www.accessdata.fda.gov/scripts/cder/daf/index.cfm?event=overview.process&varApplNo=761049)	Oncology	CD274 (PD-L1)
**Belinostat** (http://www.accessdata.fda.gov/scripts/cder/daf/index.cfm?event=overview.process&varApplNo=206256)	Oncology	UGT1A1
**Binimetinib** (http://www.accessdata.fda.gov/scripts/cder/daf/index.cfm?event=overview.process&varApplNo=210498)	Oncology	BRAF<br>UGT1A1
**Blinatumomab** (http://www.accessdata.fda.gov/scripts/cder/daf/index.cfm?event=overview.process&varApplNo=125557)	Oncology	BCR-ABL1 (Philadelphia chromosome)
**Bosutinib** (http://www.accessdata.fda.gov/scripts/cder/daf/index.cfm?event=overview.process&varApplNo=203341)	Oncology	BCR-ABL1 (Philadelphia chromosome)

**Brentuximab Vedotin** (http://www.accessdata.fda.gov/scripts/cder/daf/index.cfm?event=overview.process&varApplNo=125388)	Oncology	ALK
**Brigatinib** (http://www.accessdata.fda.gov/scripts/cder/daf/index.cfm?event=overview.process&varApplNo=208772)	Oncology	ALK
**Busulfan** (http://www.accessdata.fda.gov/scripts/cder/daf/index.cfm?event=overview.process&varApplNo=09386)	Oncology	BCR-ABL1 (Philadelphia chromosome)
**Cabozantinib** (http://www.accessdata.fda.gov/scripts/cder/daf/index.cfm?event=overview.process&varApplNo=203756)	Oncology	RET
**Capecitabine** (http://www.accessdata.fda.gov/scripts/cder/daf/index.cfm?event=overview.process&varApplNo=020896)	Oncology	DPYD
**Ceritinib** (http://www.accessdata.fda.gov/scripts/cder/daf/index.cfm?event=overview.process&varApplNo=205755)	Oncology	ALK
**Cetuximab** (http://www.accessdata.fda.gov/scripts/cder/daf/index.cfm?event=overview.process&varApplNo=125084)	Oncology	EGFR<br>RAS
**Cisplatin** (http://www.accessdata.fda.gov/scripts/cder/daf/index.cfm?event=overview.process&varApplNo=018057)	Oncology	TPMT
**Cobimetinib** (http://www.accessdata.fda.gov/scripts/cder/daf/index.cfm?event=overview.process&varApplNo=206192)	Oncology	BRAF
**Crizotinib** (http://www.accessdata.fda.gov/scripts/cder/daf/index.cfm?event=overview.process&varApplNo=202570)	Oncology	ALK<br>ROS1
**Dabrafenib** (http://www.accessdata.fda.gov/scripts/cder/daf/index.cfm?event=overview.process&varApplNo=202806)	Oncology	BRAF<br>G6PD<br>RAS
**Dasatinib** (http://www.accessdata.fda.gov/scripts/cder/daf/index.cfm?event=overview.process&varApplNo=021986)	Oncology	BCR-ABL1 (Philadelphia chromosome)
**Denileukin Diftitox** (http://www.accessdata.fda.gov/scripts/cder/daf/index.cfm?event=overview.process&varApplNo=103767)	Oncology	IL2RA<br>(CD25 antigen)
**Dinutuximab** (http://www.accessdata.fda.gov/scripts/cder/daf/index.cfm?event=overview.process&varApplNo=125516)	Oncology	MYCN

**Durvalumab** (http://www.accessdata.fda.gov/scripts/cder/daf/index.cfm?event=overview.process&varApplNo=761069)	Oncology	CD274 (PD-L1)
**Enasidenib** (http://www.accessdata.fda.gov/scripts/cder/daf/index.cfm?event=overview.process&varApplNo=209606)	Oncology	IDH2
**Encorafenib** (http://www.accessdata.fda.gov/scripts/cder/daf/index.cfm?event=overview.process&varApplNo=210496)	Oncology	BRAF
**Erlotinib** (http://www.accessdata.fda.gov/scripts/cder/daf/index.cfm?event=overview.process&varApplNo=021743)	Oncology	EGFR
**Everolimus** (http://www.accessdata.fda.gov/scripts/cder/daf/index.cfm?event=overview.process&varApplNo=022334)	Oncology	ERBB2 (HER2)<br>ER
**Exemestane** (http://www.accessdata.fda.gov/scripts/cder/daf/index.cfm?event=overview.process&varApplNo=020753)	Oncology	ER, PgR
**Fluorouracil** (http://www.accessdata.fda.gov/scripts/cder/daf/index.cfm?event=overview.process&varApplNo=012209)	Oncology	DPYD
**Fulvestrant** (http://www.accessdata.fda.gov/scripts/cder/daf/index.cfm?event=overview.process&varApplNo=021344)	Oncology	ERBB2 (HER2)<br>ER, PgR
**Gefitinib** (http://www.accessdata.fda.gov/scripts/cder/daf/index.cfm?event=overview.process&varApplNo=206995)	Oncology	EGFR<br>CYP2D6
**Ibrutinib** (http://www.accessdata.fda.gov/scripts/cder/daf/index.cfm?event=overview.process&varApplNo=205552)	Oncology	BTK<br>Chromosome 17p deletion<br>Chromosome 11q deletion
**Imatinib** (http://www.accessdata.fda.gov/scripts/cder/daf/index.cfm?event=overview.process&varApplNo=021588)	Oncology	KIT<br>BCR-ABL1 (Philadelphia chromosome)<br>PDGFRB<br>FIP1L1-PDGFRA
**Inotuzumab Ozogamicin** (http://www.accessdata.fda.gov/scripts/cder/daf/index.cfm?event=overview.process&varApplNo=761040)	Oncology	BCR-ABL1 (Philadelphia chromosome)
**Irinotecan** (http://www.accessdata.fda.gov/scripts/cder/daf/index.cfm?event=overview.process&varApplNo=020571)	Oncology	UGT1A1

**Lapatinib** (http://www.accessdata.fda.gov/scripts/cder/daf/index.cfm?event=overview.process&varApplNo=022059)	Oncology	ERBB2 (HER2)<br>ER, PgR<br>HLA-DQA1, HLA-DRB1
**Letrozole** (http://www.accessdata.fda.gov/scripts/cder/daf/index.cfm?event=overview.process&varApplNo=020726)	Oncology	ER, PgR
**Mercaptopurine** (http://www.accessdata.fda.gov/scripts/cder/daf/index.cfm?event=overview.process&varApplNo=09053)	Oncology	TPMT
**Mercaptopurine** (http://www.accessdata.fda.gov/scripts/cder/daf/index.cfm?event=overview.process&varApplNo=205919)	Oncology	NUDT15
**Midostaurin** (http://www.accessdata.fda.gov/scripts/cder/daf/index.cfm?event=overview.process&varApplNo=207997)	Oncology	FLT3<br>NPM1<br>KIT
**Neratinib** (http://www.accessdata.fda.gov/scripts/cder/daf/index.cfm?event=overview.process&varApplNo=208051)	Oncology	ERBB2 (HER2)<br>ER, PgR
**Nilotinib** (http://www.accessdata.fda.gov/scripts/cder/daf/index.cfm?event=overview.process&varApplNo=022068)	Oncology	BCR-ABL1 (Philadelphia chromosome)<br>UGT1A1
**Niraparib** (http://www.accessdata.fda.gov/scripts/cder/daf/index.cfm?event=overview.process&varApplNo=208447)	Oncology	BRCA
**Nivolumab** (http://www.accessdata.fda.gov/scripts/cder/daf/index.cfm?event=overview.process&varApplNo=125554)	Oncology	BRAF<br>CD274 (PD-L1)<br>Microsatellite Instability, Mismatch Repair
**Obinutuzumab** (http://www.accessdata.fda.gov/scripts/cder/daf/index.cfm?event=overview.process&varApplNo=125486)	Oncology	MS4A1<br>(CD20 antigen)
**Olaparib** (http://www.accessdata.fda.gov/scripts/cder/daf/index.cfm?event=overview.process&varApplNo=206162)	Oncology	BRCA
**Olaratumab** (http://www.accessdata.fda.gov/scripts/cder/daf/index.cfm?event=overview.process&varApplNo=761038)	Oncology	PDGFRA
**Omacetaxine** (http://www.accessdata.fda.gov/scripts/cder/daf/index.cfm?event=overview.process&varApplNo=203585)	Oncology	BCR-ABL1 (Philadelphia chromosome)

**Osimertinib** (http://www.accessdata.fda.gov/scripts/cder/daf/index.cfm?event=overview.process&varApplNo=208065)	Oncology	EGFR
**Palbociclib** (http://www.accessdata.fda.gov/scripts/cder/daf/index.cfm?event=overview.process&varApplNo=207103)	Oncology	ER<br>ERBB2 (HER2)
**Panitumumab** (http://www.accessdata.fda.gov/scripts/cder/daf/index.cfm?event=overview.process&varApplNo=125147)	Oncology	EGFR<br>RAS
**Pazopanib** (http://www.accessdata.fda.gov/scripts/cder/daf/index.cfm?event=overview.process&varApplNo=022465)	Oncology	UGT1A1<br>HLA-B
**Pembrolizumab** (http://www.accessdata.fda.gov/scripts/cder/daf/index.cfm?event=overview.process&varApplNo=125514)	Oncology	BRAF<br>CD274 (PD-L1)<br>Microsatellite Instability, Mismatch Repair
**Pertuzumab** (http://www.accessdata.fda.gov/scripts/cder/daf/index.cfm?event=overview.process&varApplNo=125409)	Oncology	ERBB2 (HER2)<br>ER, PgR
**Ponatinib** (http://www.accessdata.fda.gov/scripts/cder/daf/index.cfm?event=overview.process&varApplNo=203469)	Oncology	BCR-ABL1 (Philadelphia chromosome)
**Rasburicase** (http://www.accessdata.fda.gov/scripts/cder/daf/index.cfm?event=overview.process&varApplNo=103946)	Oncology	G6PD<br>CYB5R
**Ribociclib** (http://www.accessdata.fda.gov/scripts/cder/daf/index.cfm?event=overview.process&varApplNo=209092)	Oncology	ER, PgR<br>ERBB2 (HER2)
**Rituximab** (http://www.accessdata.fda.gov/scripts/cder/daf/index.cfm?event=overview.process&varApplNo=103705)	Oncology	MS4A1<br>(CD20 antigen)
**Rucaparib** (http://www.accessdata.fda.gov/scripts/cder/daf/index.cfm?event=overview.process&varApplNo=209115)	Oncology	BRCA<br>CYP2D6<br>CYP1A2
**Tamoxifen** (http://www.accessdata.fda.gov/scripts/cder/daf/index.cfm?event=overview.process&varApplNo=017970)	Oncology	ER, PgR<br>F5<br>(Factor V Leiden)<br>F2 (Prothrombin)
**Thioguanine** (http://www.accessdata.fda.gov/scripts/cder/daf/index.cfm?event=overview.process&varApplNo=012429)	Oncology	TPMT<br>NUDT15

**Trametinib** (http://www.accessdata.fda.gov/scripts/cder/daf/index.cfm?event=overview.process&varApplNo=204114)	Oncology	BRAF<br>G6PD<br>RAS
**Trastuzumab** (http://www.accessdata.fda.gov/scripts/cder/daf/index.cfm?event=overview.process&varApplNo=103792)	Oncology	ERBB2 (HER2)<br>ER, PgR
**Tretinoin** (http://www.accessdata.fda.gov/scripts/cder/daf/index.cfm?event=overview.process&varApplNo=020438)	Oncology	PML-RARA
**Vemurafenib** (http://www.accessdata.fda.gov/scripts/cder/daf/index.cfm?event=overview.process&varApplNo=202429)	Oncology	BRAF<br>RAS
**Venetoclax** (http://www.accessdata.fda.gov/scripts/cder/daf/index.cfm?event=overview.process&varApplNo=208573)	Oncology	Chromosome 17p deletion

[a]: Representative biomarkers are listed based on standard nomenclature as per the Human Genome Organization (HUGO) symbol and/or simplified descriptors using other common conventions.
[b]: ER: estrogen receptor.
[c]: PgR: progesterone receptor.

https://www.fda.gov/drugs/scienceresearch/ucm572698.htm

Supplementary Table 2: NGS platforms applications.

	Whole genome sequencing							
	Large Whole-Genome Sequencing	Small Whole-Genome Sequencing	Exome sequencing	Targeted Gene Sequencing	Metagenomic	Transcriptome	Methylation Sequencing	ChipSeq
**ABI/SOLiD**	no	no	Yes	Yes	Yes	Yes	Yes	Yes
**Illumina**								
NovaSeq 6000	Yes	Yes	Yes	Yes	Yes	Yes	Yes	Yes
HiSeq X Series	Yes	no	no	no	no	no	no	no
HiSeq 4000 System	Yes	Yes	Yes	Yes	Yes	Yes	Yes	Yes
NextSeq Series	Yes	Yes	Yes	Yes	Yes	Yes	Yes	Yes
MiSeq Series	no	Yes	no	Yes	Yes	no	no	Yes
MiniSeq System	no	Yes	no	Yes	Yes	no	no	no
iSeq 100 System	no	no	no	Yes	no	no	no	no
**Ion Torrent**								
Ion GeneStudio S5	no	Yes	Yes	Yes	Yes	Yes	no	Yes
Ion Proton	no	Yes	Yes	Yes	Yes	Yes	no	Yes
Ion PGM	no	Yes	no	Yes	no	no	no	no
**Pacific Biosystems**	Yes	Yes	Yes	Yes	Yes	Yes	Yes	Yes
**Oxford nanopore**	Yes	Yes	Yes	Yes	Yes	Yes	Yes	Yes
**BGI500SEQ**	Yes	Yes	Yes	Yes	Yes	Yes	Yes	Yes

Supplementary Table 3. Strategy, technology and performance of NGS platforms.

Platform	Instrument	Output per Run	Maximum Read Length	Read per Run	Run Time	Error Bias	Sequencing Technology	Library amplification	Advantages	Limitations
ABI/SOLiD [a]	SOLiD 5500 W	160–260 Gb	75 bp	3 B	5–8 d	mismatch	Sequencing by ligation	EmPCR	High throughput	Cumbersome emPCR; long run time
	SOLiD 5500 xl W	160–320 Gb	75 bp	6 B	4–8 d	mismatch				
Illumina [a]	NovaSeq 6000 System	65–3000 Gb	2 × 150 bp	1.3–20 B	13–44 h	mismatch	Reversible terminator sequencing by synthesis	Bridge amplification	Very high throughput	Run and read length; high investment for the instrument
	HiSeq X Series	1.6–1.8 Tb	2 × 150 bp	5.3–6 B	< 3 d	mismatch				
	HiSeq 4000 System	105–1500 Gb	2 × 150 bp	2.1 M–5 B	<1–3.5 d	mismatch				
	NextSeq Series	25–120 Gb	2 × 150 bp	130–400 M	12–30 h	mismatch				
	MiSeq Series	540 Mb–15 Gb	2 × 300 bp	1-25 M	4–55 h	mismatch				
	MiniSeq System	1.65–7.5 Gb	2 × 150 bp	7–25M	4–24 h	mismatch				
	iSeq 100 System	144–1.2 Gb	2 × 150 bp	4 M	9–17.5 h	mismatch				
Ion Torrent [a]	Ion PGM	100 Mb–2 Gb	400 bp	0.6–5.5 M	2–7 h	indel	Sequencing by synthesis	EmPCR	Read length; short run time	Increased errors rate by multiple PCR steps
	Ion Proton	10–32 Gb	200 bp	82–330 M	2–4 h	indel				
	Ion GeneStudio S5	0.5–50 Gb	600 bp	2–130 M	3–24 h	indel				
Pacific Biosystems [b]	PacBio RSII	500 Mb–1 Gb	13500 bp	560-660 M	0.5–4 h	indel	Single-molecule, real-time DNA sequencing by synthesis	No PCR	Long reads; short run time	Reads per run low; high error rates
	Sequel	5–10 Gb	10000 bp	350 M	8 h	N/A				
Oxford Nanopore [b]	MInION	1–5 Gb	100 kb	100 M	1–2 d	indel mismatch	Nanopore exonuclease sequencing	No PCR	Long reads; low cost	High error rates
BGI Group [b]	BGI500SEQ	20 Gb	100		24 h	N/A	Probe-Anchor Synthesis (cPAS) and DNA Nanoballs (DNB)	Rolling circle replication	High throughput; low error rate	Read length

Abbreviations: M=million; B=billion; h=hours; d=days
(a) Second generation sequencing
(b) Third generation sequencing

Supplementary Table 4: Reference examples of hotspot, actionable and disease-focused panels.

Company	KIT	n. of genes	Mutation type	Pathology	Instrument
Illumina	AmpliSeq for Illumina BRCA Panel	2	Somatic and germline	Breast and ovarian cancer	iSeq 100 System<br>MiniSeq System<br>MiSeq System
	Cancer Hotspot Panel v2	50	Somatic	Lung, colon, breast, ovarian, melanoma and prostate cancer	iSeq 100 System<br>MiniSeq System<br>MiSeq System
	AmpliSeq for Illumina Comprehensive Panel v3	161	Somatic	Lung, colon, breast, bladder, pancreatic, ovarian, melanoma and prostate cancer	NextSeq 550 System
	AmpliSeq for Illumina Comprehensive Cancer Panel	409	Somatic	Lung, colon, breast, ovarian, melanoma, and prostate cancer	NextSeq 550 System
	TruSight® Tumor 170	170	Somatic	Lung, colon, breast, bladder, pancreatic, gastric, ovarian, melanoma and prostate cancer	HiSeq 2500 System<br>NextSeq 550 System
	TruSight Oncology 500	523	Somatic	Colon, endometrium, gastric, lung, melanoma and uterine cancer	NextSeq 550 System
	AmpliSeq for Illumina Myeloid Panel	24	Germline	Myeloid disorders	MiniSeq System<br>MiSeq System
	TargetRich™ PGxComplete	43	Germline	Antidepressants, ADHD, statins, platelet aggregation inhibitors, anticoagulents drug classes	MiniSeq System<br>MiSeq System<br>HiSeq 2500 System<br>NextSeq 550 System
ThermoFisher	Oncomine BRCA Research Assay	2	Somatic and germline	Breast and ovarian cancer	Ion PGM System<br>Ion S5 System
	Oncomine Focus Assay	52	Somatic	Lung, colon, breast, bladder, pancreatic, ovarian, melanoma and prostate cancer	Ion S5 System
	Oncomine Comprehensive Assay v3	161	Somatic	Lung, colon, breast, bladder, pancreatic, gastric, ovarian, melanoma and prostate cancer	Ion S5 System
	Oncomine Myeloid Research Assay	29	Germline	Myeloid disorders	Ion PGM System<br>Ion S5 System
	Ion AmpliSeq Pharmacogenomics Research Panel	40	Germline	Antidepressants, ADHD, statins, platelet aggregation inhibitors, anticoagulents drug classes	Ion S5 System
